# High-Oxygen Submersion Fetal Thymus Organ Cultures Enable FOXN1-Dependent and -Independent Support of T Lymphopoiesis

**DOI:** 10.3389/fimmu.2021.652665

**Published:** 2021-03-30

**Authors:** Jianxun Han, Juan Carlos Zúñiga-Pflücker

**Affiliations:** ^1^ Department of Biological Sciences, Sunnybrook Research Institute, Toronto, ON, Canada; ^2^ Department of Immunology, University of Toronto, Toronto, ON, Canada

**Keywords:** FOXN1, Delta-like 4, T cell development, FTOC, oxygen, thymus epithelial cells

## Abstract

T cell development is effectively supported in fetal thymus organ cultures (FTOCs), which places thymus lobes atop an air-liquid interface (ALI) culture system. The direct exposure to air is critical for its success, as fetal thymus lobes placed in low oxygen submersion (LOS)-FTOCs fail to support thymocyte development. However, submersion cultures performed in the presence of high concentration of ambient oxygen (60~80%) allow for normal thymocyte development, but the underlying mechanism for this rescue has remained elusive. Here, we show that FOXN1 expression in thymic epithelial cells (TECs) from LOS-FTOCs was greatly reduced compared to conventional ALI-FTOCs. Consequently, the expression of important FOXN1 target genes, including *Dll4* and *Ccl25*, in TECs was extinguished. The loss of DLL4 and CCL25 interrupted thymocyte differentiation and led to CD4^+^CD8^+^ cells exiting the lobes, respectively. High oxygen submersion (HOS)-FTOCs restored the expression of FOXN1 and its target genes, as well as maintained high levels of MHCII expression in TECs. In addition, HOS-FTOCs promoted the self-renewal of CD4^−^CD8^−^CD44^−^CD25^+^ cells, allowing for the continuous generation of later stage thymocytes. Forced FOXN1 expression in TECs rescued thymocyte developmental progression, but not cellularity, in LOS-FTOCs. Given that oxidative stress has been reported to accelerate the onset of age-associated thymic involution, we postulate that regulation of FOXN1 by oxygen and antioxidants may underpin this biological process.

## Introduction

T cell development is an intricate process that requires the thymus microenvironment, especially thymic epithelial cells (TECs), which provide signals to promote progenitor cells to differentiate towards the T-lineage (1). FOXN1 is an indispensable master transcriptional regulator for the development and differentiation of TECs. FOXN1 also regulates the expression of many TEC genes that are essential to induce and support T cell development within the thymus (2). For example, *Dll4* is a direct transcriptional target of FOXN1 and DLL4-mediated Notch signaling is absolutely required for thymocyte development (2–4). CCL25 and CXCL12, two chemokines whose expression are regulated by FOXN1 (2), are known to facilitate homing of thymic seeding progenitors (TSPs) to thymus, and intrathymic trafficking and differentiation of developing thymocytes (5).

Differentiation of progenitor cells with multi-lineage potential towards the T-lineage is conventionally classified into four stages based on CD4 and CD8 expression. The earliest thymocytes do not express either CD4 or CD8, termed double negative (DN) cells, which first give rise to CD8^+^ immature single positive (ISP) cells and then CD4^+^CD8^+^ double positive (DP) cells. After successful selection events, DP cells differentiate into either CD4 or CD8 single positive (SP) T cells. DN cells can be further classified based on the expression of CD44 and CD25, as CD44^+^CD25^−^ DN1, CD44^+^CD25^+^ DN2, CD44^-^CD25^+^ DN3, and CD44^−^CD25^−^ DN4 stages.

The first method to culture mouse fetal thymuses employed an air-liquid interface (ALI) system (6). This ALI culture system was later developed into the currently widely used fetal thymus organ culture (FTOC), in which thymus lobes from embryonic gestation day 12 to 15 (E12 ~ E15) are placed onto a Nuclepore filter that rests on top of a surgical Gelfoam sponge soaked with culture medium (7). The direct exposure to air was critical for its success, as low oxygen submersion (LOS) of fetal thymus lobes in culture medium failed to effectively support thymocyte differentiation compared to ALI-FTOCs (8–10). On the other hand, placing fetal thymus lobes in high oxygen (~70% O_2_) submersion (HOS)-FTOCs allowed for T cell development, and rescued both cellularity and differentiation progression towards conventional αβ T-lineage cells (10). However, how increased oxygen availability promoted αβ T cell development in submersion cultures remains unknown.

Here, we found that FOXN1 levels, and consequently the expression of its direct transcriptional targets, such as *Dll4* and *Ccl25*, are rapidly reduced in TECs from LOS-FTOCs, as compared to ALI-FTOCs, and could be partially restored by HOS-FTOCs. Additionally, restoring FOXN1 protein levels by forced expression in LOS cultures resulted in the emergence of TCRβ-expressing CD4^+^ and CD8^+^ SP cells without affecting total cellularity. Furthermore, we observed that increased oxygen availability could promote self-renewal of DN3 cells to augment the cellularity of submersion FTOCs. Taken together, expression of FOXN1 appears to be regulated by oxidative states that dictate thymic epithelial cell function and hence T cell development.

## Materials and Methods

### Mice

All mice were bred and housed in the Comparative Research Facility at Sunnybrook Research Institute under specific pathogen-free conditions. All animal procedures were approved by Sunnybrook Research Institute Animal Care Committee and performed in accordance with the committee’s ethical standards.

R26Foxn1 mouse strain was a kind gift from Dr. Clare Blackburn (University of Edinburgh, UK), and it differs from the previously reported R26Foxn1ER strain (11) only in that a non-fused FOXN1 protein, rather than fused FOXN1ER^T2^ protein, is expressed when upstream *loxp/STOP/loxp* sequence is removed by Cre recombinase. Foxn1^ex9cre^ [B6(Cg)-*Foxn1^tm3(cre)Nrm^*/J, stock #: 018448], R26-CreER^T2^ [B6.129-*Gt(ROSA)26Sor^tm1(cre/ERT2)Tyj^*/J, Stock #: 008463], RAG2 deficient [B6(Cg)-*Rag2^tm1.1Cgn^*/J, stock #: 008449], Vav-iCre (B6.Cg-*Commd10^Tg(Vav1-icre)A2Kio^*/J, stock # 008610), ROSA26-rtTA-IRES-EGFP [B6.Cg-*Gt(ROSA)26Sor^tm1(rtTA,EGFP)Nagy^*/J, stock #: 005670] strains were all purchased from The Jackson Laboratory (12–16). Vav-iCre mice were bred to ROSA26-rtTA-IRES-EGFP mice to generate mice whose thymocytes express EGFP. Timed-pregnant CD1 females were purchased from Charles River Laboratories. For in-house timed-mating, female and male mice were housed together overnight and then separated next morning. Thymus lobes from embryos 14 days after separation, considered as E14.5, were used for all experiments.

### Air-Liquid Interface (ALI)- and Submersion FTOC

To set up conventional ALI-FTOC, individual thymus lobes were place on a Whatman^®^ Nuclepore™ Track-Etched membrane (Cat. # WHA110409 from Sigma-Aldrich) that was placed on top of SURGIFORM^®^ absorbable gelatin sponge (Cat. # 1974 from Ethicon) in wells with 1.5 ml of DMEM containing 10% fetal bovine serum in a 12-well plate. For submersion FTOC, individual thymus lobes were placed in wells with 0.2 ml of culture medium in non-tissue culture treated 96-well plates (Falcon^®^ 351177), which avoids stromal cells from attaching and spreading onto plate surface, in order to maintain thymus lobe architecture. The 96-well plates were either placed inside of a cell culture incubator with 5% CO_2_ balanced with air, as low oxygen submersion (LOS) culture, or sealed inside of a plastic bag filled with 70% O_2_, 5% CO_2_, and 25% of N_2_, as high oxygen submersion (HOS) culture. For LOS culture with tamoxifen, 4-OHT was added to the cultures at a final concentration of 5 nM. Unless indicated otherwise, fetal thymus lobes from the same timed-pregnant females were used for different experiments so that each culture in any assay represented an independent biological replicate.

### Flow Cytometric Analysis

To examine the developmental stages of thymocytes, cells outside of lobes were collected directly by transferring the culture medium with cells to a 1.5-ml microcentrifuge tube, and cells inside of lobes were released by squeezing lobes against 70 μm nylon mesh with a syringe plunger. Live cells were counted using hemocytometer and stained with the following panel of antibodies: AF700/CD45 (clone 30-F11, BioLegend), PE-Cy7/CD8a (clone 53-6.7, BioLegend), APC/CD4 (clone GK1.5, in-house conjugated), APC-Cy7/CD25 (clone PC61, BioLegend), PerCp-Cy5.5/CD44 (clone IM7, BioLegend), BV510/CD3 (clone 145-2C11, BioLegend), FITC/TCRβ (clone H57-597, in-house conjugated), and PE/γδTCR (clone GL3, BD Biosciences). FITC/CD11b (clone M1/70, BioLegend) and FITC-CD19 (clone MB19-1, BioLegend) were included in the analysis of *Rag2*-deficient thymocytes. Data were acquired using BD-LSR II and analyzed with FlowJo.

To measure thymocyte proliferation and apoptosis, cultures were incubated with 20 μM of EdU (ThermoFisher Scientific) at 37°C for an hour before harvest. Cells inside of the lobes from one ALI-FTOC culture, 3 HOS cultures, 4 LOS, or 4 iFoxN1-LOS cultures were pooled together. After Fc block and surface staining with AF700/CD45 (clone 30-F11, BioLegend), PE-Cy7/CD8a (clone 53-6.7, BioLegend), APC/CD4 (clone GK1.5, in-house conjugated), APC-Cy7/CD25 (clone PC61, BioLegend), PerCp-Cy5.5/CD44 (clone IM7, BioLegend), and PE/γδTCR (clone GL3, BD Biosciences), each sample was split into two halves, with one half incubated with CellEvent Caspase 3/7 Green Reagent (ThermoFisher Scientific) at 37°C for 30 min to detect apoptotic cells and the other half continuing with EdU staining by Click-iT Plus Edu AF488 Flow Cytometry Assay kit (ThermoFisher Scientific) after fixable live/dead eFluor 450 staining (ThermoFisher Scientific) to detect cells at S phase of a cell cycle.

To estimate TEC numbers and subtypes, cultures were enzymatically disassociated using 1X TrypLE (ThermoFisher Scientific) at 37°C for 20 min followed by pipetting (~10 times) until lobes were fully disassociated. Live cells were counted using hemocytometer and, after Fc block, were stained with PerCp-Cy5.5/CD45 (clone 30-F11, eBioscience), PE-Cy7/CD326 (clone G8.8, BioLegend), PE/Ly-51 (clone 6C3, BioLegend), AF647/CD205 (clone NLDC-145, BioLegend), APC-Cy7/Streptavidin/Biotinylated UEA-1 (BD Bioscence), and AF700/MHCII (I-A/I-E, clone M5/114.15.2, BioLegend). TEC cellularity was estimated by multiplying the numbers of total live cells to the percentage of CD45^-^EpCam^+^ cells among single live cells from each culture.

To measure the expression levels of DLL4, MHCII, and FOXN1 in TECs, cells were enzymatically disassociated in the same way as above followed by staining with APC/DLL4 [clone YW152F, Genentech (17), in-house conjugated], or APC-Cy7/MHCII (I-A/I-E, clone M5/114.15.2, BioLegend), together with AF700/CD45 (clone 30-F11 from BD Biosciences) and PE/CD326 (clone G8.8, eBioscience). For FOXN1 intracellular staining, samples pre-stained with APC/DLL4 were incubated with fixable live/dead eFluor 450 (ThermoFisher Scientific) on ice for 30 min, fixed with 4% paraformaldehyde at room temperature for 10 min, permeabilized with pre-chilled (−20°C) methanol for 5 min, then incubated with a mouse monoclonal anti-FOXN1 antibody (18), a kind gift from Dr. Hans-Reimer Rodewald (dkfz, Heidelberg, Germany), for 30 min followed by incubation with PE-Cy7-conjugated anti-mouse IgG2b secondary antibody (clone RMG2b-1, Biolegend). For flow cytometric analysis of *Dll4* and *Foxn1* mRNA levels in TECs, PrimeFlow™ RNA assay kit and type I mouse *Dll4* or *Foxn1* target probe (ThermoFisher Scientific) was used.

### Quantitative PCR (qPCR)

Individual cultures were lysed in 0.5 ml of Trizol (ThermoFisher Scientific) for RNA preparation and cDNA was then synthesized using LunaScript RT SuperMix kit (New England Biolabs). Expression levels of *Il7*, *Scf*, and *Ccl25* were quantified by qPCR using Luna Universal qPCR kit in a QuantStudio™ 5 PCR system (ThermoFisher Scientific) and keratin 8 (*Krt8*) was used as reference gene to normalize the epithelial content within the inputs. Data were analyzed with DA2 App (ThermoFisher Scientific).

### Statistical Analysis

Statistical analysis as indicated in figure legends were performed using GraphPad Prism 9 graphing and statistics software. Depending on the data distribution, a log-transformation was applied for the analysis of data shown using log scales. Not significant (ns) p > 0.05; *p < 0.05; **p < 0.01; ***p < 0.001; ****p < 0.0001.

## Results

### FOXN1 Expression in TECs Is Regulated by Oxygen Availability

**Figure 1:**
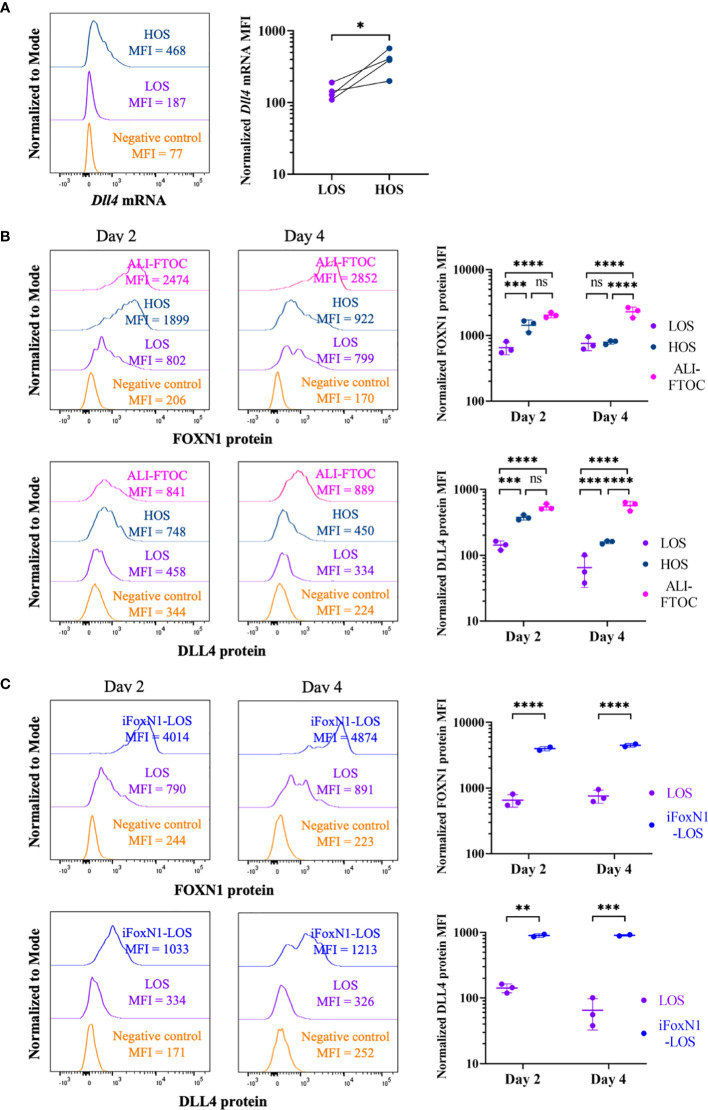
Regulation of *FoxN1* and *Dll4* expression by oxygen availability. **(A)** The left panel shows representative histogram overlays of *Dll4* transcript levels using PrimeFlow RNA assay and the right panel shows scatter dot plots and statistical analysis (ratio paired Student’s t-test) of the mean fluorescence intensity (MFI) of *Dll4* transcript, as indicated. **(B)** Flow cytometric analysis of intracellular FOXN1 and cell surface DLL4 expression in TECs from LOS-, HOS-, and ALI-FTOC, as indicated. Representative histogram overlays and statistical analysis (two-way ANOVA with *post-hoc* Tukey’s test) are shown for FOXN1 (top panels) and DLL4 (bottom panels) protein expression in TECs from day 2 and day 4 cultures. **(C)** Flow cytometric analysis of intracellular FOXN1 and cell surface DLL4 expression in TECs from LOS and iFoxN1-LOS cultures, as indicated. Samples were pre-gated on CD45^-^EpCam^+^ cells except for negative control, which were CD45^+^ cells. Numbers of biologically independent trials are indicated. ns, not significant; p > 0.05; *p < 0.05; **p < 0.01; ***p < 0.001; ****p < 0.0001.

To address the effect of low oxygen submersion (LOS)-FTOCs on TEC function, we first examined whether the expression of the key Notch ligand, *Dll4*, is altered, as previously shown in thymic stromal monolayer cell cultures (19). After a 2-day period in LOS culture conditions, *Dll4* expression in TECs was lost, while in high oxygen (70% O_2_) submersion (HOS) culture *Dll4* expression in a significant fraction of TECs was largely maintained (**Figure 1A** and **Supplementary Figure 1A**). Since *Dll4* is a direct transcriptional target of FOXN1 (2), we investigated whether the changes in *Dll4* expression levels were due to alternations in FOXN1 levels. Flow cytometric analysis revealed that while FOXN1 protein levels in TECs from LOS-FTOCs were lower than that from ALI-FTOCs, increasing oxygen availability in HOS cultures restored FOXN1 protein levels in TECs after a 2-day culture period (**Figure 1B**). This modulation of *Foxn1* expression by oxygen availability occurred at transcription level (**Supplementary Figure 1B**). However, the difference in FOXN1 protein levels between HOS and LOS conditions became less apparent when cultures were extended to day 4, at which point FOXN1 protein levels in TECs from HOS-FTOCs decreased. Cell surface expression of DLL4 in TECs from day 4 HOS cultures was also reduced but still detectable. To address whether the reduction of FOXN1 expression, and its target genes, between days 2 and 4 in HOS cultures may be due to the growth of developing thymocytes, which would restrict oxygen diffusion inside lobes and/or directly affect FOXN1 levels *via* thymocyte-stroma crosstalk (18), we performed cultures with fetal thymuses from *Rag2*-deficient mice. Of note, FOXN1 and DLL4 expression in TECs from *Rag2^−/−^* HOS-FTOCs retained higher levels than those from corresponding LOS-FTOCs throughout 9 days of culture (**Supplementary Figure 1C**), suggesting that a potential mechanism for HOS cultures to support T cell development is the maintenance of both FOXN1 and DLL4 expression.

### Forced FOXN1 Expression Rescues Thymocyte Development, but Not Cellularity, in LOS Cultures

To investigate whether FOXN1 mediates the rescue of thymocyte development by increased oxygen availability in HOS, we took advantage of the R26Foxn1 transgenic mice developed by Dr. Blackburn’s group, in which a mouse *Foxn1* expression cassette, preceded by a *loxp-STOP-loxp* element, was inserted into the *Rosa 26* locus. When this strain was bred to Foxn1^ex9cre^ mice, developed by Dr. Manley’s group, all TECs from the resulting E14.5 thymus lobes that had expressed endogenous *Foxn1* maintained high levels of FOXN1 protein, and its target DLL4, even when cultured as LOS-FTOCs (**Figure 1C**, iFoxN1-LOS). These results show that ectopic expression of FOXN1 in LOS cultures can rescue DLL4 expression. Although exposing thymus lobes from E14.5 embryos of R26Foxn1^creERT2^ mice to tamoxifen could also induce FOXN1 expression (**Supplementary Figure 1D**, LOS-4OHT), this approach raised the concern that exposing thymocytes to tamoxifen and potential FOXN1 induction in non-TEC cells might have undesired side effects. Therefore, we withheld exposure to tamoxifen to lobes from E14.5 embryos of R26Foxn1^creERT2^ mice, which were used as control cultures.

**Figure 2:**
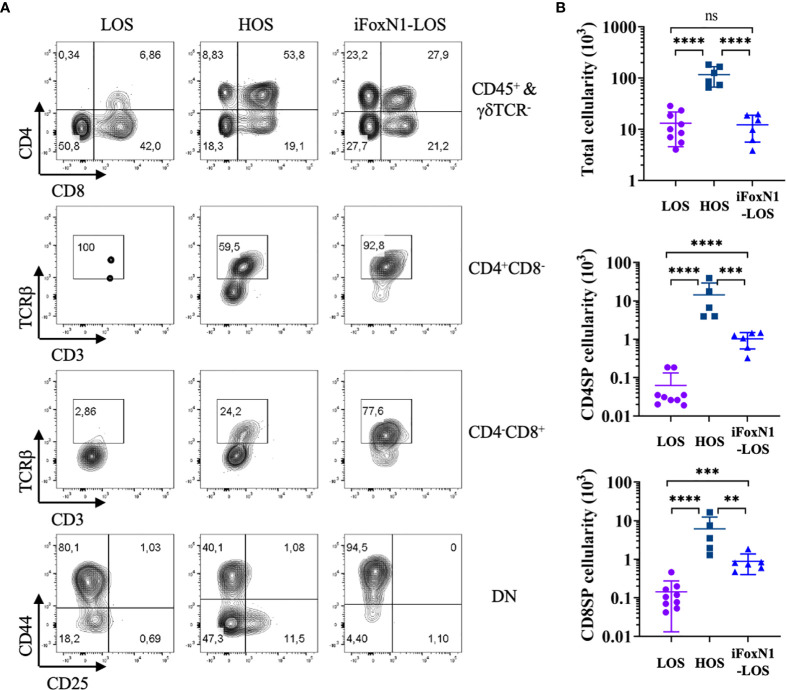
Generation of αβ T cells in HOS- and iFoxN1-LOS-FTOC. **(A)** Flow cytometric analysis of thymocytes from 8-day LOS, HOS, and iFoxN1-LOS cultures, as indicated. The top panels display CD4 and CD8 expression of CD45^+^γδTCR^-^ gated cells. The middle two panels display TCRβ and CD3 expression of CD4^+^CD8^−^ gated cells, and CD4^−^CD8^+^ gated cells, respectively. The bottom panels display CD44 and CD25 expression of CD4^−^CD8^−^ DN cells. **(B)** Scatter dot plots and statistical analysis (one-way ANOVA with *post-hoc* Tukey’s test) of total (top), CD4SP (middle), and CD8SP (bottom) cell numbers from 8-day cultures, as indicated. ns, not significant; p > 0.05; **p < 0.01; ***p < 0.001; ****p < 0.0001.

Consistent with previous studies (8–10), after 8 days of culture, few DP cells and TCRβ-expressing CD4SP or CD8SP cells emerged from LOS-FTOCs, as compared to their presence in HOS-FTOCs (**Figure 2A**). An 8-fold increase in total cellularity was obtained from HOS-FTOCs, including 230- and 40-fold increase in CD4SP and CD8SP cells, respectively, as compared to control LOS-FTOC (**Figure 2B**). Notably, DP cells and TCRβ-expressing CD4SP and CD8SP cells developed in iFoxN1-LOS-FTOCs (**Figure 2A**). Although there was no difference in the total cellularity between LOS and iFoxN1-LOS cultures, iFoxN1-LOS-FTOCs produced 16- and 6-fold more CD4SP and CD8SP cells, respectively, than LOS-FTOCs (**Figure 2B**), demonstrating that enforced FOXN1 expression in TECs could rescue T lymphopoiesis in LOS cultures, but yielding much lower total cellularity than HOS-FTOCs. We tested whether iFoxN1 lobes in HOS cultures would be affected by enforced FOXN1 expression and found HOS cultures showed increased total cellularity and the numbers of CD4SP and CD8SP. In addition, DP cells remained as the dominant subpopulation, the same as the HOS cultures of lobes without forced FOXN1 expression. Therefore, the significant lower cellularity from iFoxN1-LOS compared to HOS cultures was not due to very high levels of FOXN1 proteins and its target genes in TECs (**Supplementary Figure 2**).

### Oxygen Availability Influences TEC Cellularity and Thymopoietic Activity

TECs provide necessary cues to promote thymocyte survival and proliferation in addition to guide their commitment and differentiation towards the T-lineage. The notable difference in total cellularity between FTOCs from HOS and LOS conditions, despite the rescue in thymocyte differentiation afforded by the expression of FOXN1, prompted us to investigate whether oxygen availability modulates TEC cellularity and differentiation. We recovered significantly less TECs from LOS than HOS cultures after 8 days regardless of FOXN1 protein levels (**Supplementary Figure 3A**). While no significant difference in the numbers of recovered TECs between 4-day and 8-day cultures, it was clear that there was a significant, ~5-fold, reduction in the numbers of TECs that could be recovered during the first 4 day of culture from lobes without forced FOXN1 expression (**Supplementary Figures 3A, B**), suggesting a loss of TECs in LOS cultures or that LOS culture condition made it difficult to recover TECs for flow cytometric analysis. Furthermore, HOS cultures had a higher thymopietic index, the ratio of thymocytes to TECs (20), indicating TECs from HOS cultures were functionally more capable of supporting thymocyte survival and/or proliferation (**Supplementary Figure 3A**). No consistent difference in the expression of common subtype markers was observed between LOS and HOS cultures although cultures with forced FOXN1 expression showed higher level of CD205, which is a direct FOXN1 target gene (**Supplementary Figures 3A, C**) (2).

To test whether the failure of TECs to support thymocyte growth and differentiation in LOS cultures is reversible, we switched FTOCs from LOS to HOS condition after 8 days of culture and concomitantly added fetal liver lineage-negative Sca1^+^Kit^+^ (LSK) cells. The cultures were analyzed 16 days after the switch, with HOS condition showing robust cellularity and differentiation potential, as evidenced by the presence of DP cells, in stark contrast to cultures that remained as LOS conditions (**Supplementary Figure 4**). These findings demonstrate that defects of TECs in LOS cultures is reversible.

### IL-7 and SCF Levels Are Not Affected by Oxygen Availability

The difference in thymopoietic activity between TECs from HOS and LOS cultures urged us to examine two important lymphopoietic cytokines, IL-7, and SCF, whose deficiency leads to lower thymic cellularity in knockout mice (21–25). qPCR analysis revealed no difference in *Il7* transcript levels between HOS and LOS culture conditions (**Supplementary Figure 5**). In addition, no significant difference was noted in *Scf* transcript levels between HOS and LOS FTOCs. This suggests that it is unlikely that increased oxygen availability in HOS led to higher cellularity through elevating cytokine levels.

### Increased Oxygen Availability in HOS Cultures Promotes Initial Rapid Growth

**Figure 3:**
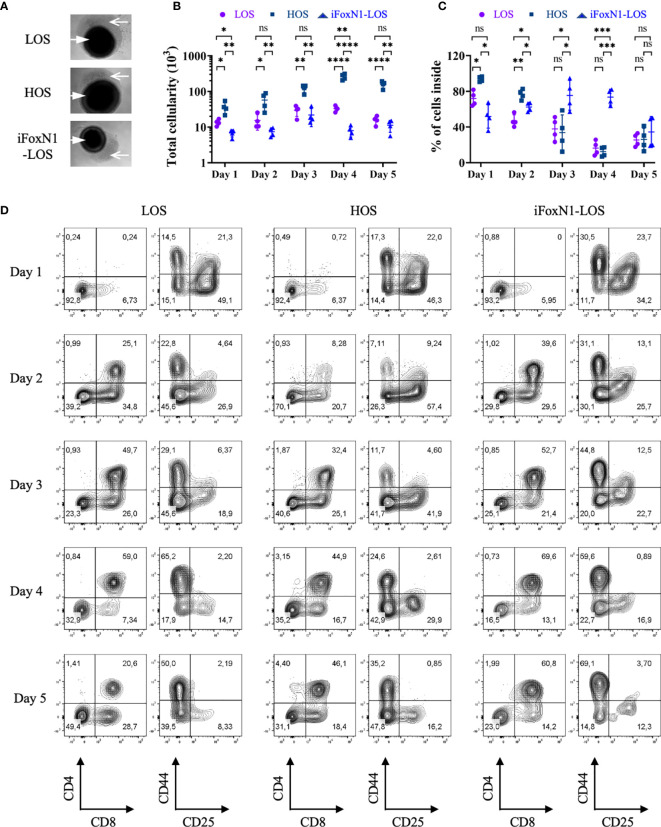
Temporal analysis of T cells development from LOS, HOS, and iFoxN1-LOS cultures. **(A)** Phase-contrast images of submersion cultures on day 2 of culture are shown, with closed arrow pointing to thymus lobes and open arrow indicating cells outside of lobes. **(B)** Scatter dot plots and statistical analysis (two-way ANOVA with *post-hoc* Tukey’s test) of total cell numbers, including cells both inside and outside of lobes, of individual culture conditions on each day within the first 5 days of culture. **(C)** Scatter dot plots and statistical analysis (two-way ANOVA with *post-hoc* Tukey’s test) of the percentage (%) of cells inside of lobes, as indicated. **(D)** Flow cytometric analysis of thymocytes inside of lobes from LOS, HOS, and iFoxN1-LOS cultures, as indicated. For each culture condition, the left contour plots show CD4 and CD8 expression of CD45^+^γδTCR^−^ gated cells and the right panels display CD44 and CD25 expression of CD45^+^γδTCR^−^CD4^−^CD8^−^ gated cells. ns, not significant; p > 0.05; *p < 0.05; **p < 0.01; ***p < 0.001; ****p < 0.0001.

We next examine the temporal kinetics of thymocyte cellularity and differentiation during early period of submersion cultures. Since cells were found present outside of the lobes (**Figure 3A**), we analyzed cells inside and outside of lobes separately, with the consideration that cells outside of the thymus might develop differently due to a lack of direct support from TECs. There was negligible cell growth within the first two days of LOS cultures as the total numbers of cells, including cells both outside and inside of lobes, were slightly lower than the numbers of cells (1.5–2.0 × 10^4^) of the starting E14.5 thymus lobes (**Figure 3B**), consistent with a previous report (9). Of note, a large fraction of cells in LOS cultures migrated out of lobes during the first two days (**Figure 3C**). In contrast, HOS cultures promoted thymocyte growth from the start of culture, yielding a ~3-fold increase in cellularity compared to LOS-FTOCs on day 1, increasing to 4-fold by day 2 (**Figure 3B**). In addition, most cells in HOS cultures remained inside of lobes (**Figure 3C**), which would ensure direct support from TECs. Further examination of thymocyte proliferation and apoptosis rates revealed that increased proliferation, rather than decreased apoptosis, was the driving force behind the initial rapid increase in total cellularity in HOS cultures (**Supplementary Figure 6**).

Notwithstanding the stark differences in total cellularity and cell location, all three cultures showed similar initial developmental progression (**Figure 3D**), with appearance of CD8^+^ ISP on day 1 and DP cells on day 2. The seemingly low percentage of DP cells in HOS on day 2 was due to relatively higher numbers of DN and CD8^+^ ISP, as the number of DP was comparable to, if not higher than, the other two cultures (**Supplementary Figure 7**). The LOS-FTOCs showed an increase in the frequency of DP cells up to day 4, which then collapsed by day 5 of culture (**Figure 3B**). This was in contrast to HOS and iFoxN1-LOS cultures, which maintained high percentages of DPs, and, in the case of HOS-FTOCs, higher DP cellularity, by day 5 of culture.

### Enhanced Self-Renewal of DN3 Cells in HOS-FTOC

Notably, the numbers of DP cells inside both LOS and iFoxN1-LOS cultures peaked on day 3 and then declined (**Supplementary Figure 7**), consistent with a single wave of DP cells being generated. In contrast, the numbers of DP cells inside HOS-FTOCs remained relatively constant after day 3, even until day 8 of culture, suggesting a continuous generation of DP cells. These findings were consistent with the notable persistence of DN3 cells seen in HOS-FTOCs, but not in LOS cultures (**Figures 2A**, **3D**).

**Figure 4:**
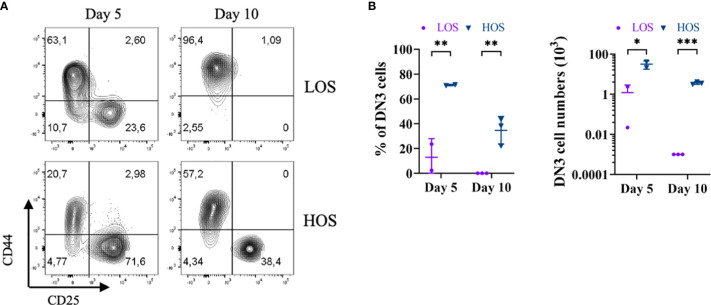
Increased oxygen availability promotes DN3 self-renewal in HOS. **(A)** Flow cytometric analysis of *Rag2*-deficient thymocytes from 5- and 10-day LOS and HOS cultures, as indicated. Cells shown were pre-gated as CD45^+^CD11b^−^CD19^−^CD4^−^CD8^−^. **(B)** Scatter dot plots and statistical analysis (two-way ANOVA with *post-hoc* Sidak’s test) of percentage (%) of DN3 cells (left), and numbers of DN3 cells (right), as indicated. DN3 cells were not detected in LOS cultures on day 10, as such were indicated and analyzed as one cell to enable log-transformation. *p < 0.05; **p < 0.01; ***p < 0.001.

To test whether increased oxygen availability could promote the expansion and/or self-renewal of DN3 cells in HOS-FTOCs, we cultured E14.5 *Rag2* ^−/−^ thymus lobes in submersion cultures. As shown in **Figure 4**, by day 5 of culture, the number and frequency of DN3 cells are severely reduced in LOS as compared to HOS FTOCs. By day 10 of culture, there were few, if any, DN3 cells left in LOS, while HOS cultures contained ~40% DN3 cells, suggesting that DN3 cellularity is maintained under HOS conditions.

### Failure to Retain DP cells Inside and Down-Regulation of MHCII in LOS Cultures

Consistent with previous work (8–10), TCRβ^+^ CD4SP and CD8SP cells were not generated in LOS-FTOC (**Figure 2A**). Hence, the DP cells present early on in LOS cultures (**Figure 3D**) somehow fail to give rise SP cells, while iFoxN1-LOS-FTOCs allowed for the generation of TCRβ^+^ SP cells (**Figure 2A**). One outstanding difference between LOS and iFoxN1-LOS cultures was the location of DP cells; while most DP cells in iFoxN1-LOS remained inside of the lobes, most DP cells in LOS cultures were found outside of the lobes, where they would not likely receive support to undergo positive selection (**Supplementary Figure 7**).

**Figure 5:**
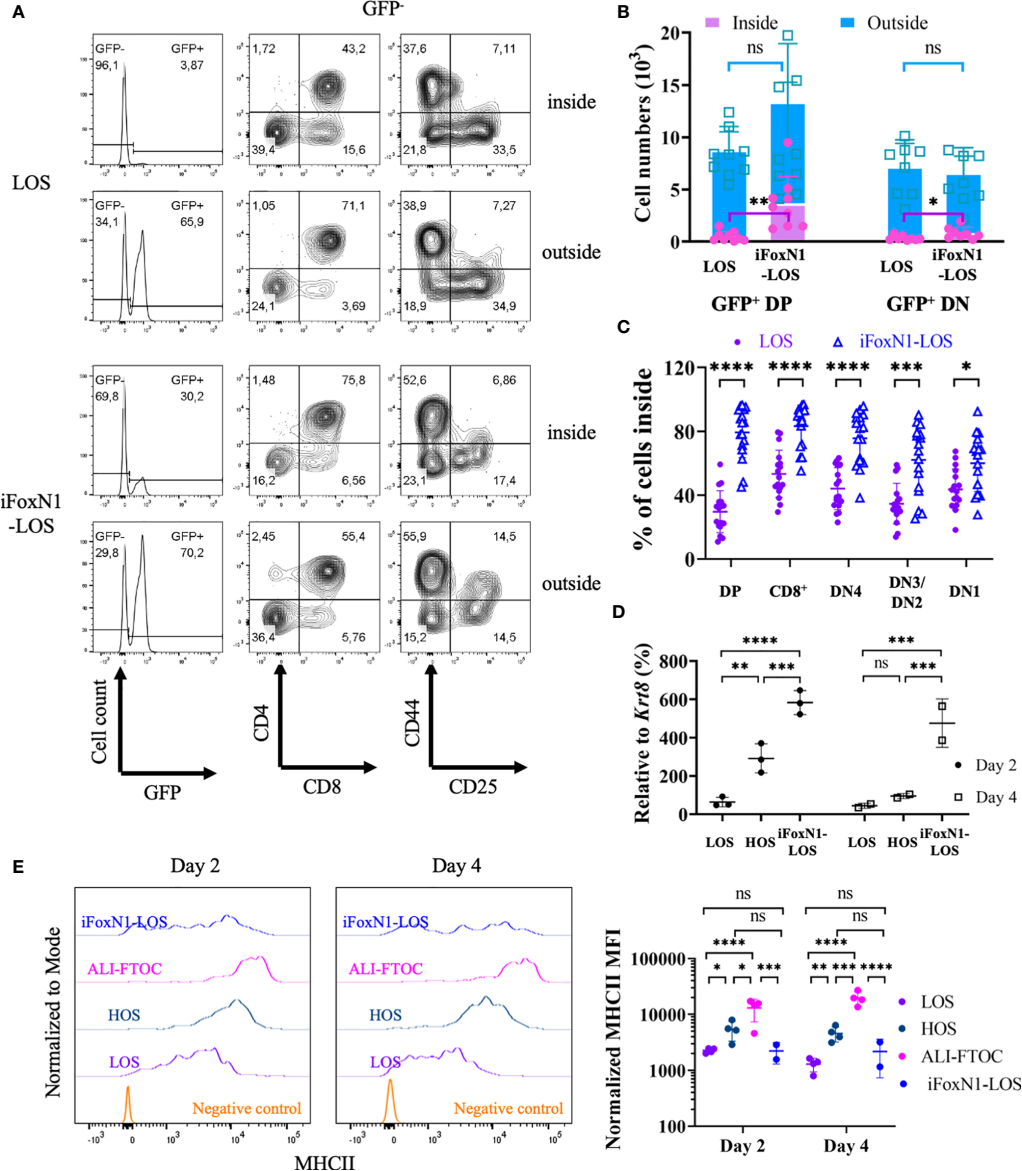
Migration of CD4^+^CD8^+^ cells in LOS cultures. **(A)** Flow cytometric analysis of cells inside and outside of lobes from LOS- and iFoxN1-LOS-FTOCs. Sorted GFP-expressing CD4^+^CD8^+^ cells were added to LOS-FTOCs and all cells were analyzed after 1-day of culture. The left panels show histograms of GFP expression from CD45^+^γδTCR^−^ gated cells. The middle and right panels show CD4 and CD8 expression on CD45^+^γδTCR^−^GFP^−^ cells, and CD44 and CD25 expression on CD45^+^γδTCR^−^GFP^−^CD4^−^CD8^−^ cells, respectively. **(B)** Stacked column graphs and statistical analysis (unpaired Student’s t-test) of numbers of added GFP^+^ CD4^+^CD8^+^ (DP), or CD4^−^CD8^−^ DN2/DN3 (DN) cells, inside and outside of lobes after 1-day of culture, as indicated. **(C)** Scatter dot plots and statistical analysis (unpaired Student’s t-test) of percentage (%) of endogenous (GFP^-^) cells in different subsets that remained inside of the lobes after 1-day of culture, as indicated. **(D)** Scatter dot plots and statistical analysis (two-way ANOVA with *post-hoc* Tukey’s test) of *Ccl25* transcript levels relative to keratin 8 (*Krt8*) quantified by RT-qPCR, as indicated. **(E)** Flow cytometric analysis of MHCII expression on TECs from LOS-, HOS-, ALI-, and iFoxN1-LOS-FTOCs. Representative histogram overlays and statistical analysis (two-way ANOVA with *post-hoc* Tukey’s test) of MHCII levels on day 2 and day 4 of cultures are shown, as indicated. Samples were pre-gated on CD45^-^EpCam^+^ cells except for negative control, which were CD45^+^ cells. ns, not significant; p > 0.05; *p < 0.05; **p < 0.01; ***p < 0.001; ****p < 0.0001.

To test whether LOS-FTOCs are less capable of attracting/maintaining DP cells inside, thymus lobes free of unattached cells were transferred to new wells together with sorted GFP^+^ DP or DN2/DN3 cells, distinguishing GFP^−^ endogenous from GFP^+^ exogenous thymocytes. Cells outside or inside of the lobes were collected, counted and analyzed separately. Both GFP^−^ and GFP^+^ cells could be detected inside and outside of the lobes, with GFP^−^ cells outside of the lobes being endogenous cells that emigrated within the 1-day culture period (**Figure 5A**). It was clear that more GFP^+^ DP cells were attracted into iFoxN1-LOS than LOS lobes. As a result, more exogenous GFP^+^ DP cells were present inside the lobes of iFoxN1-LOS than LOS cultures (**Figure 5B**). In contrast, a significant fraction (~70%) of endogenous GFP^−^ DP cells emigrated from LOS-FTOCs, while iFoxN1-LOS cultures maintained most (~80%) of endogenous GFP^−^ DP cells inside of the lobes (**Figure 5C**). In addition, iFoxN1-LOS-FTOCs seemed to attract/maintain all subpopulations inside of the lobes better than LOS cultures (**Figure 5C**). To address a potential molecular mechanism responsible for the difference in attracting/maintaining DP cells inside iFoxN1-LOS, as opposed to LOS-FTOCs, we examined the expression of *Ccl25*, a direct FOXN1 target gene (2) and a chemoattractant of CCR9^+^ DP cells. Our analysis revealed that after a 2-day culture, *Ccl25* transcript levels were higher in HOS and iFoxN1-LOS-FTOCs compared to LOS cultures, and remained higher in iFoxN1-LOS-FTOCs after a 4-day of culture (**Figure 5D**).

Additionally, LOS cultures, not only failed to keep DP cells from emigrating, but also showed lower levels of MHCII expression in TECs compared to HOS-FTOCs (**Figure 5E** and **Supplementary Figure 3C**). The expression of MHCII on TECs was found to be dependent on oxygen availability and independent of FOXN1, as control HOS conditions maintained high levels of MHCII on day 4, when FOXN1 levels were down-regulated (**Figure 1**). In addition, about half of the TECs from iFoxN1-LOS cultures expressed very low levels of MHCII. The low levels of MHCII in half of the TECs from iFoxN1-LOS likely affected the selection of CD4SP cells.

## Discussion

Access to adequate oxygen levels is required for T cell development within the thymic microenvironment, which is achieved by the vascularization of thymus at about E15.5 *in vivo* (26), or by direct exposure to air in ALI-FTOCs *in vitro* (7). Sufficient oxygen supply provides more than energy necessary for developing thymocytes to proliferate and survive, as immature thymocytes could still grow and respond to cytokine stimuli, but failed to develop into mature αβ T cells, when fetal thymus lobes were cultured as submersion FTOCs (8–10), an unquestionable hypoxic environment due to limitations in oxygen diffusion (27). Increasing oxygen concentration in the air surrounding submersion FTOCs from 20% ambient to 60–80% was shown to alleviate the hypoxic environment and allowed for normal thymocyte development, although the molecular mechanism(s) remained elusive (10). Here, we show that increased oxygen availability in HOS cultures enabled intrathymic T lymphopoiesis through both FOXN1-dependent and -independent processes.

We found that access to oxygen was required for TECs to maintain FOXN1 expression levels and the expression of FOXN1 target genes, such as *Ccl25* and *Dll4* (2). We addressed the biological significance of maintaining high expression levels of CCL25 in submersion FTOCs, and showed that low levels of CCL25 in LOS-FTOC allowed for DP cells to emigrate from the lobes. Consequently, DP cells would lose MHC-mediated support from TECs to undergo positive selection. In HOS-FTOC, *Ccl25* expression was initially maintained, but then decreased as FOXN1 levels wane at later time points, with a subsequent migration of DP cells out of the lobes. Nevertheless, there was always a considerable number of DP cells remaining inside the HOS lobes, ensuring that these cells would be able to undergo positive selection.

On the other hand, the relevance of DLL4 during early T cell development cannot be overstated (3, 4). However, previous works (8–10), as well as this study, made use of E14.5 fetal thymuses, and, by the time DLL4 protein expression is extinguished from the cell surface of TECs in LOS-FTOCs, the majority of thymocytes are already committed to the T cell lineage, and either undergoing or already passed β-selection, two critical events that depend on Notch signaling (28, 29). Nonetheless, the continuous presence of DLL4 on TECs in HOS-FTOC likely supported a more persistent generation of DP cells, which were still the dominant subset on day 8. Consistent with this notion, previous work showed that the earlier the fetal thymus lobes are harvested, the fewer DP cells observed after 7 days of LOS cultures (9). Thus, the more severe developmental block seen with earlier fetal thymuses reflects the importance of maintaining DLL4 levels in FTOCs to enable developing thymocytes to survive β-selection.

There are potentially at least two FOXN1-independent mechanisms by which adequate oxygen supply is required to support T lymphopoiesis in submersion FTOCs: maintaining high expression levels of MHCII on TECs; and promoting self-renewal of DN3 cells. The FOXN1-independent regulation of MHCII expression by oxygen and low levels of MHCII on about half of TECs from iFoxN1-LOS-FTOCs are consistent with the previous finding that MHCII is not a direct FOXN1 target gene (2). However, it leaves open the question as to how MHCII levels remained high on the other half of TECs in iFoxN1-LOS cultures after 4 days.

Limited self-renewal of progenitor thymocytes in the absence of new TSPs has been observed *in vivo* (30, 31), and attributed to the transit amplifying DN2/DN3 subsets. It was proposed that losing competition for the DN3 niche to newly generated DN3 cells leads to the cessation of self-renewal of pre-existing DN3 cells (32), but the underlying molecular mechanism(s) has remained elusive. Submersion FTOCs might provide an excellent system to study this question as input of new TSPs can be tightly controlled. We have demonstrated that self-renewal of *Rag2* ^−/−^ DN3 cells takes place in HOS, but not in LOS cultures. Loss of DLL4 expression likely contributed to the lack of self-renewal by *Rag2* ^−/−^ DN3 cells in LOS cultures, as our previous work showed that Notch signaling promotes the survival of *Rag2* ^−/−^ DN3 cells in OP9-DL co-culture system (29). The use of the OP9-DL cell system also shows that T cell development *per se* can take place in LOS conditions, further emphasizing that reducing oxygen availability intrinsically affects TEC rather thymocyte functionality. However, whether and what other factor(s), in addition to DLL4, constitutes the intrathymic DN3 niche and how pre-existing DN3 cells lose their competitive edge remain open questions, which could be investigated using submersion FTOC system.

Adaption to hypoxia is usually accompanied with changes in the expression of hundreds of genes that are involved in a plethora of cellular activities. Therefore, it is unlikely that *Foxn1* and its target genes are the only ones whose levels were affected in LOS cultures. Nonetheless, rescue of T cell differentiation by genetically forced *Foxn1* expression demonstrates that it is one of the critical genes that mediate the regulation of T cell development by oxygen availability. Further study is needed to unveil the molecular mechanism underlying its transcriptional regulation by oxygen availability. HIF1 is the most-studied transcription factor that responds to oxygen tension to maintain cellular homeostasis under hypoxia. ChIP-Seq data demonstrated that HIF1 functions primarily as a transcription activator, as HIF1 did not bind to genes that were downregulated by HIF1 (33). Although HIF1 has been reported to directly suppress the transcription of at least two genes in nucleus pulposus cells, the stability and transcription activity of HIF1α in these cells is oddly not regulated by oxemic state (34, 35). Therefore, if HIF1 is involved in the downregulation of *Foxn1* in LOS cultures, then its effect is likely transduced by one of its target genes, rather than directly.

The physiological significance of regulating FOXN1 expression by oxygen availability remains an open question. It is noteworthy to relate the potential role of increased oxidative stress in TEC function to the findings showing that treatment with antioxidants can delay the onset of age-related thymus involution (36). The effect of antioxidants on the onset of age-related thymus involution was attributed to alleviating oxidative damage in TECs. Our observations, such as the reversibility of TEC function from LOS cultures, make us to speculate whether the effect of antioxidants on thymic involution is at least partially mediated by enabling the survival of TECs under high oxygen stress, which is required for the maintenance of FOXN1 expression. This view is also consistent with the effect of forced expression of FOXN1 in transgenic mice that appeared to rejuvenate an aged thymus (11).

In conclusion, our findings revealed that increased oxygen availability in HOS cultures restores the expressions of FOXN1 and its target genes, as well as FOXN1-independent MHCII expression, which together safeguard the normal developmental progression of conventional αβ T cells. In addition, HOS cultures promote the survival and expansion of DN3 cells, which secures a persistent supply of progenitors for β-selection and subsequent positive selection. Working together, these mechanisms enable high-oxygen support of robust T lymphopoiesis in submersion FTOCs. In addition, our results warrant further investigations on the physiological significance of FOXN1 regulation by oxygen availability and its underlying mechanisms, as well as mechanisms governing self-renewal of DN3 cells using submersion FTOCs.

## Data Availability Statement

The raw data supporting the conclusions of this article will be made available by the authors, without undue reservation.

## Ethics Statement

The animal study was reviewed and approved by Sunnybrook Research Institute Animal Care Committee.

## Author Contributions

JH designed and performed all the experiments, analyzed the data, and wrote the manuscript. JCZ-P conceived the project, analyzed the data, and wrote the manuscript. All authors contributed to the article and approved the submitted version.

## Funding

This work was supported by grants from the Natural Sciences and Engineering Research Council of Canada (NSERC, RGPIN-2016-06592), and the Canadian Institutes of Health Research (CHIR, FND-154332). JCZ-P is supported by a Canada Research Chair in Developmental Immunology.

## Conflict of Interest

The authors declare that the research was conducted in the absence of any commercial or financial relationships that could be construed as a potential conflict of interest.
