# Supplementary material for: High-Oxygen Submersion Fetal Thymus Organ Cultures Enable FOXN1-Dependent and -Independent Support of T Lymphopoiesis
Source: Front Immunol. 2021 Mar 30;12:652665. doi: 10.3389/fimmu.2021.652665 (PMC8043069; doi:10.3389/fimmu.2021.652665)
Supplement: Supplementary file 1 [file DataSheet_1.pdf]

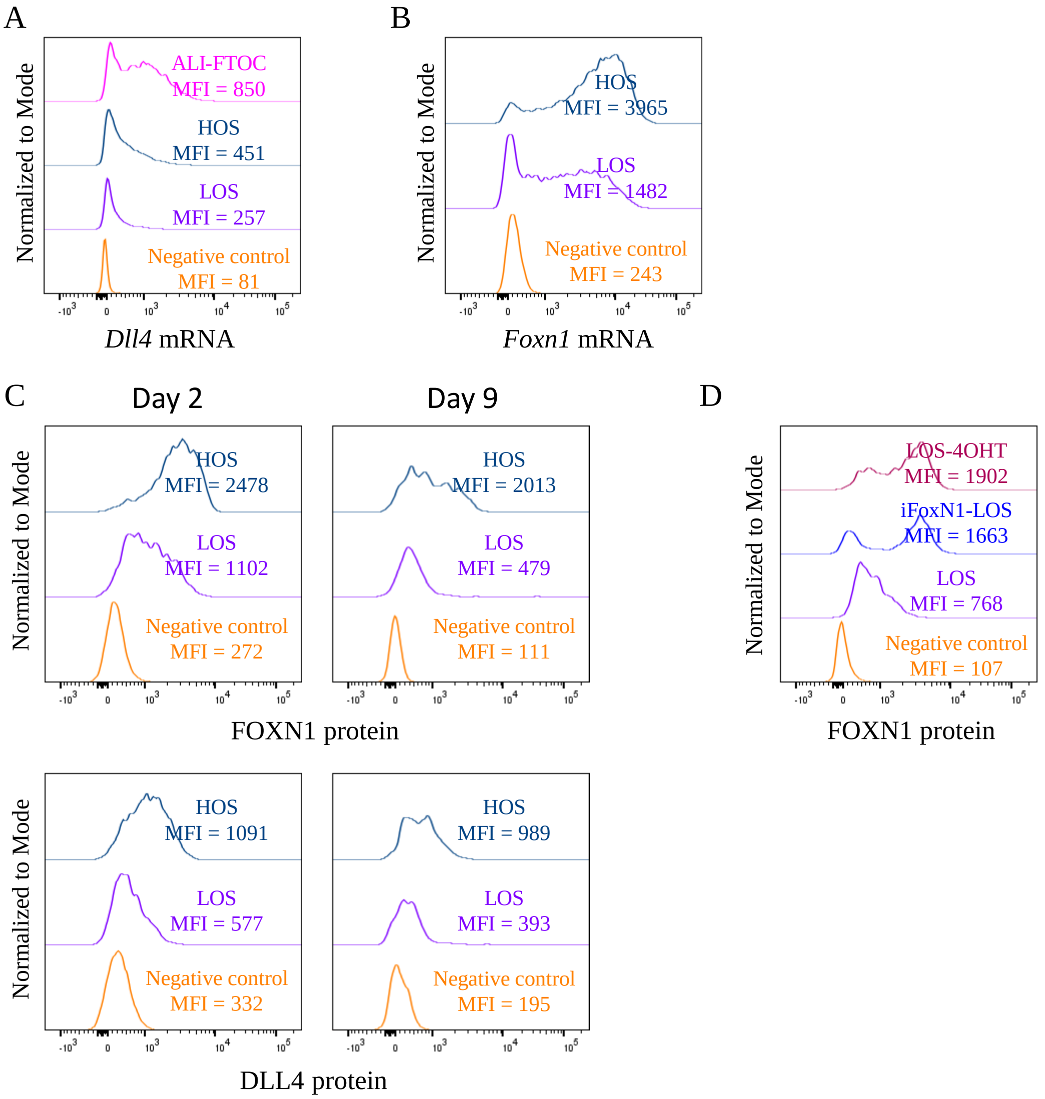

Supplemental Figure 1. Flow cytometric analysis of *Foxn1* and *Dll4* expression in TECs. (A) Flow cytometric analysis of *Dll4* mRNA from 2-day FTOC cultures. Three technical replicates from each culture condition were pooled together for this analysis. Samples were pre-gated on CD45$^-$EpCam$^+$ cells except for negative control, which were CD45$^+$ cells. (B) Flow cytometric analysis of *Foxn1* mRNA from 2-day LOS and HOS cultures of E14.5 CD1 thymus lobes. Three technical replicates from each culture condition were pooled together for this analysis. Samples were pre-gated on CD45$^-$EpCam$^+$ cells except for negative control, which were CD45$^+$ cells. (C) Flow cytometric analysis of intracellular FOXN1 and cell surface DLL4 expression in TECs from 2-day and 9-day LOS and HOS cultures of *Rag2*$^{-/-}$ E14.5 thymus lobes, as indicated. Each sample represented one culture from that condition. Samples were pre-gated on CD45$^-$EpCam$^+$ cells except for negative control, which were CD45$^+$ cells. (D) Flow cytometric analysis of intracellular FOXN1 protein levels in TECs from 2-day LOS cultures, as indicated. Three technical replicates from each culture condition were pooled together for this analysis. Samples were pre-gated on CD45$^-$EpCam$^+$ cells except for negative control, which were CD45$^+$ cells.

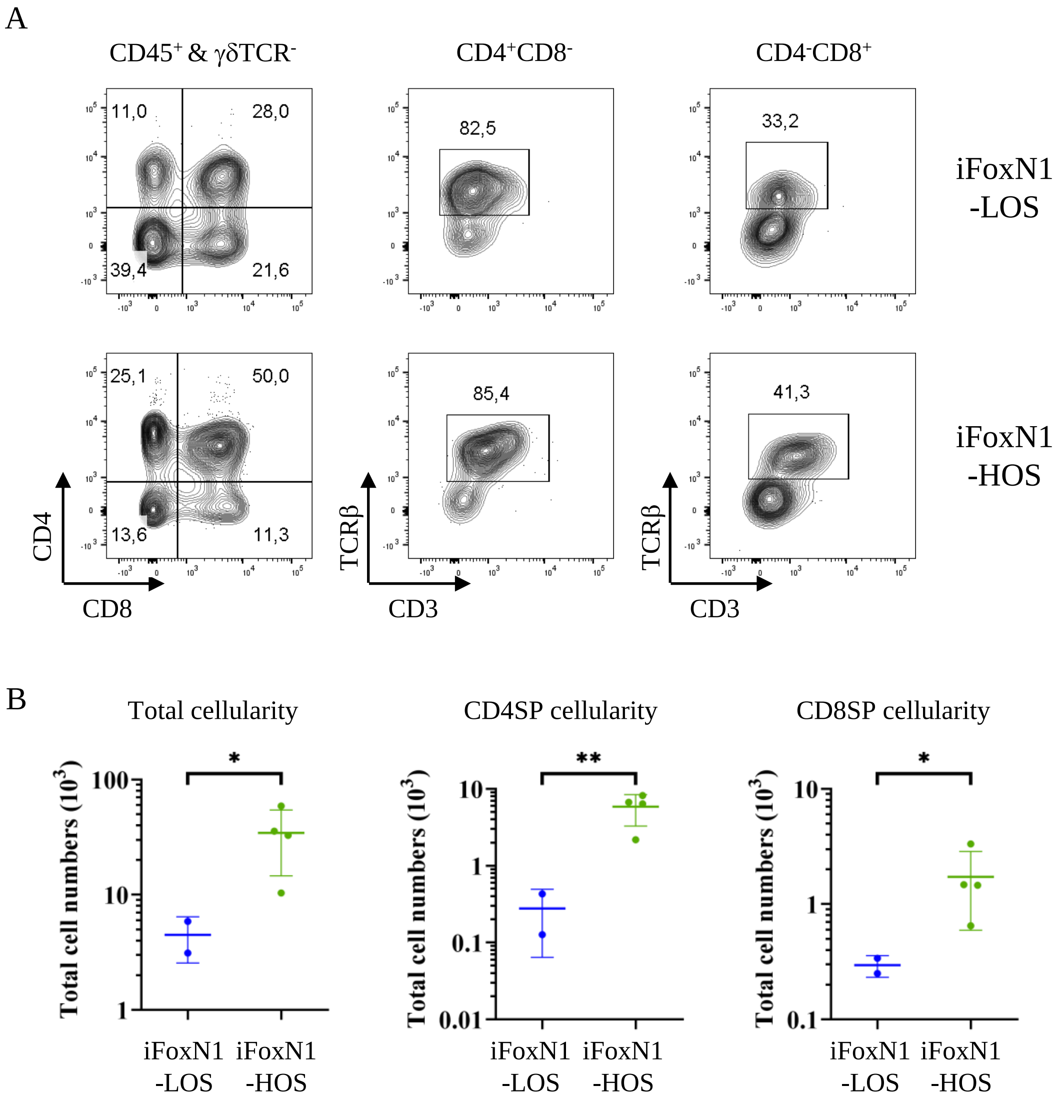

Supplemental Figure 2. Higher cellularity from iFoxN1-HOS-FTOC. (A) Flow cytometric analysis of thymocytes from 8-day iFoxN1-LOS and iFoxN1-HOS cultures, as indicated. The left panels display CD4 and CD8 expression of CD45$^+$gdTCR$^-$ gated cells. The middle panels display TCRβ and CD3 expression of CD4$^+$CD8$^-$ gated cells. The right panels display TCRβ and CD3 expression of CD4$^-$CD8$^+$ gated cells. (B) Scatter dot plots and statistical analysis (unpaired Student's t test) of total (left), CD4SP (middle) and CD8SP (right) cell numbers from 8-day cultures, as indicated. The results were from two technical replicate of iFoxN1-LOS and four technical replicates of iFoxN1-HOS cultures. *$p < 0.05$; **$p < 0.01$.

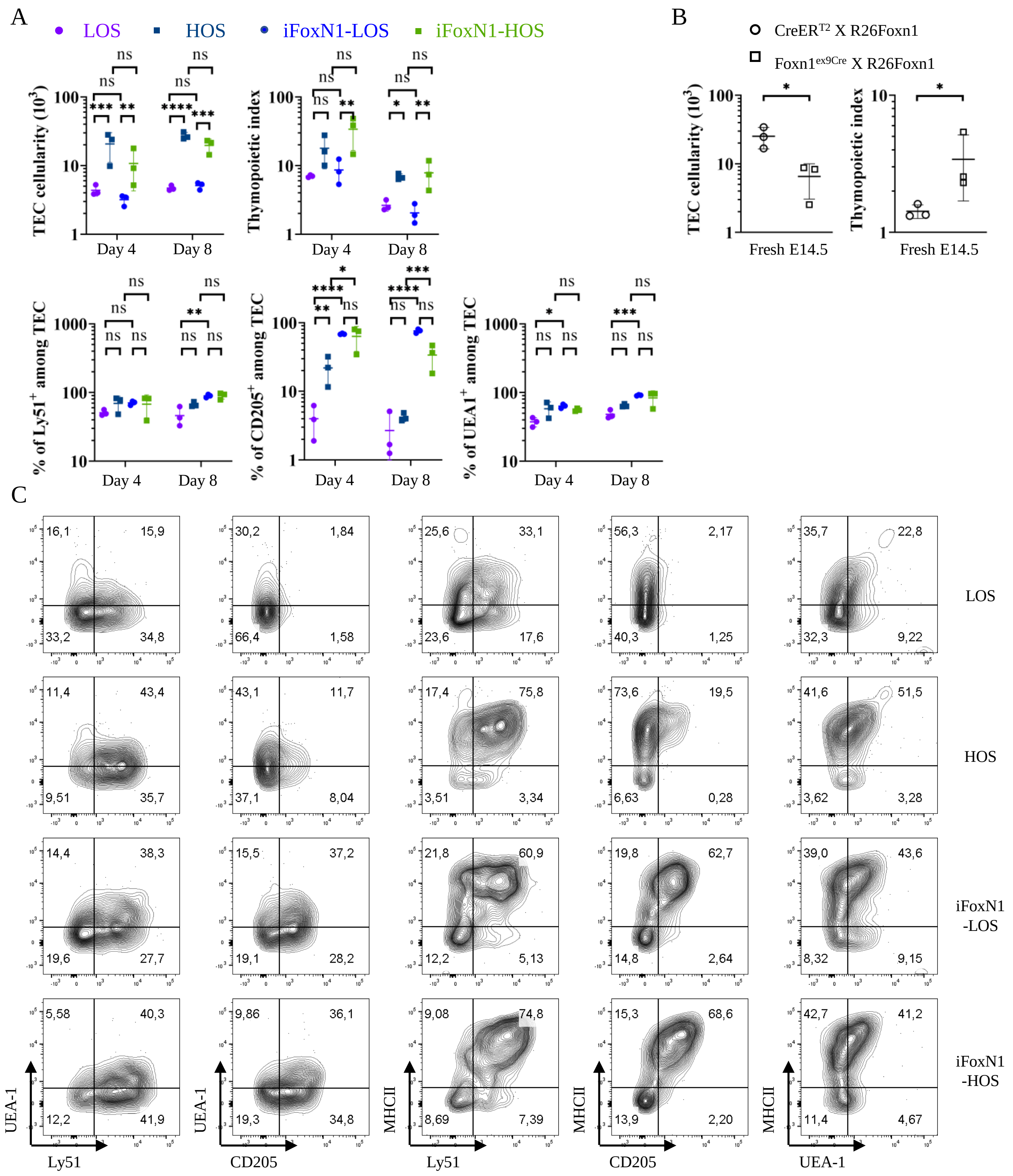

Supplemental Figure 3. TEC cellularity and subtypes. (A) Cellularity, thymopoietic index, and subtypes of TECs from 4-day and 8-day FTOC cultures, as indicated. Three technical replicates were used for the analysis. (B) Cellularity and thymopoietic index of fresh E14.5 thymus lobes from two mouse strains, as indicated. Three technical replicates were used for the analysis. (C) Flow cytometric analysis of TEC subtypes from 4-day FTOC cultures, as indicated. Samples were pre-gated on CD45$^-$EpCam$^+$ cells and the gating thresholds were determined based on CD45$^+$ cells. Not significant (ns) $p > 0.05$; $*p < 0.05$; $**p < 0.01$; $***p < 0.001$;$****p < 0.0001$.

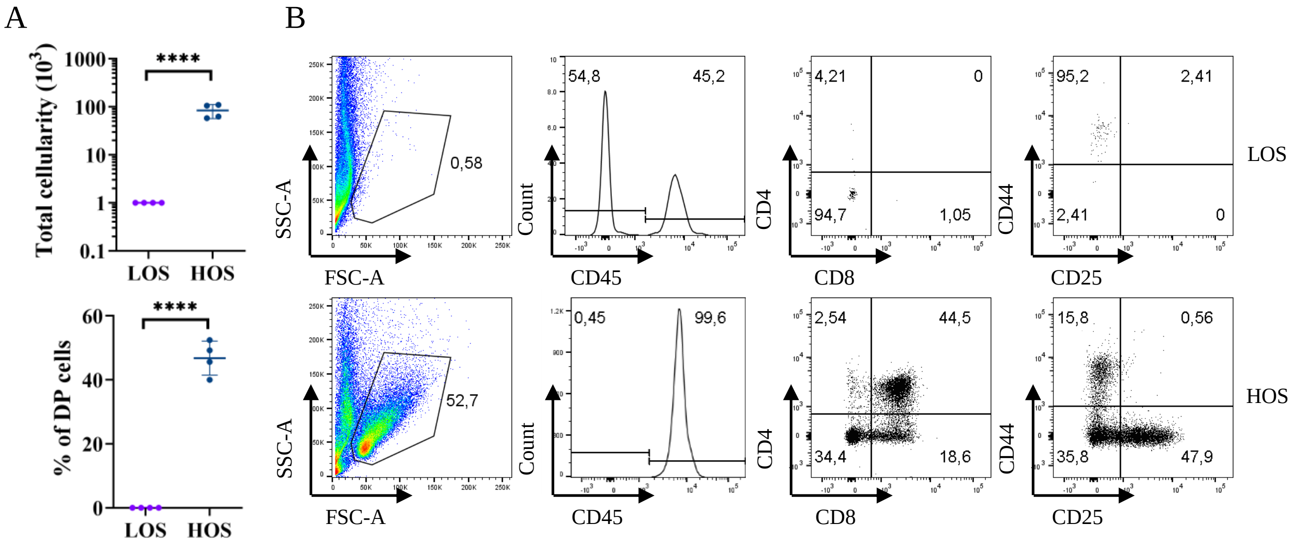

Supplemental Figure 4. Thymocyte development in LOS-converted HOS cultures. (A) Total cellularity and percentage (%) of DP population among CD45$^+$ cells from 4 technical replicate cultures 16 days after switching from LOS to HOS condition, or remaining under LOS condition, after 8 days of culture and concomitantly adding 3,000 fetal liver LSK cells, as indicated. Total cell numbers from 24-day LOS cultures were too low to be confidently counted and recorded as 1,000 cells. (B) Flow cytometric analysis of thymocytes from these cultures, as indicated. Cells from the second left panels were pre-gated on single live lymphocytes as shown in the most left panels. Cells from the third left panels were pre-gated on CD45$^+$ cells. Cells from the most right panels were pre-gated on CD4$^-$CD8$^-$ double negative cells. ****$p < 0.0001$.

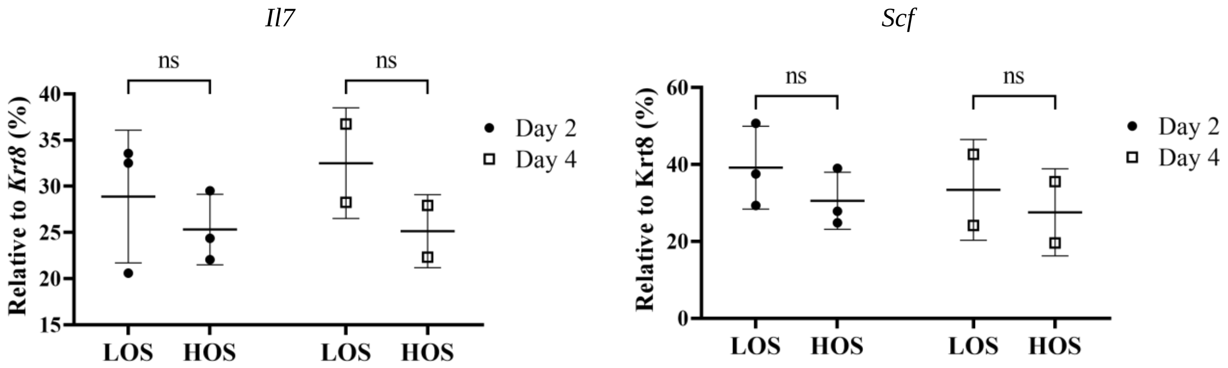

Supplemental Figure 5. Effect of oxygen availability on *Il7* and *Scf* gene expression. Scatter dot plots and statistical analysis (two-way ANOVA with post-hoc Tukey's test) of *Il7* (left) and *Scf* (right) transcript levels relative to keratin 8 (*Krt8*) quantified by RT-qPCR, as indicated. Not significant (ns) $p > 0.05$.

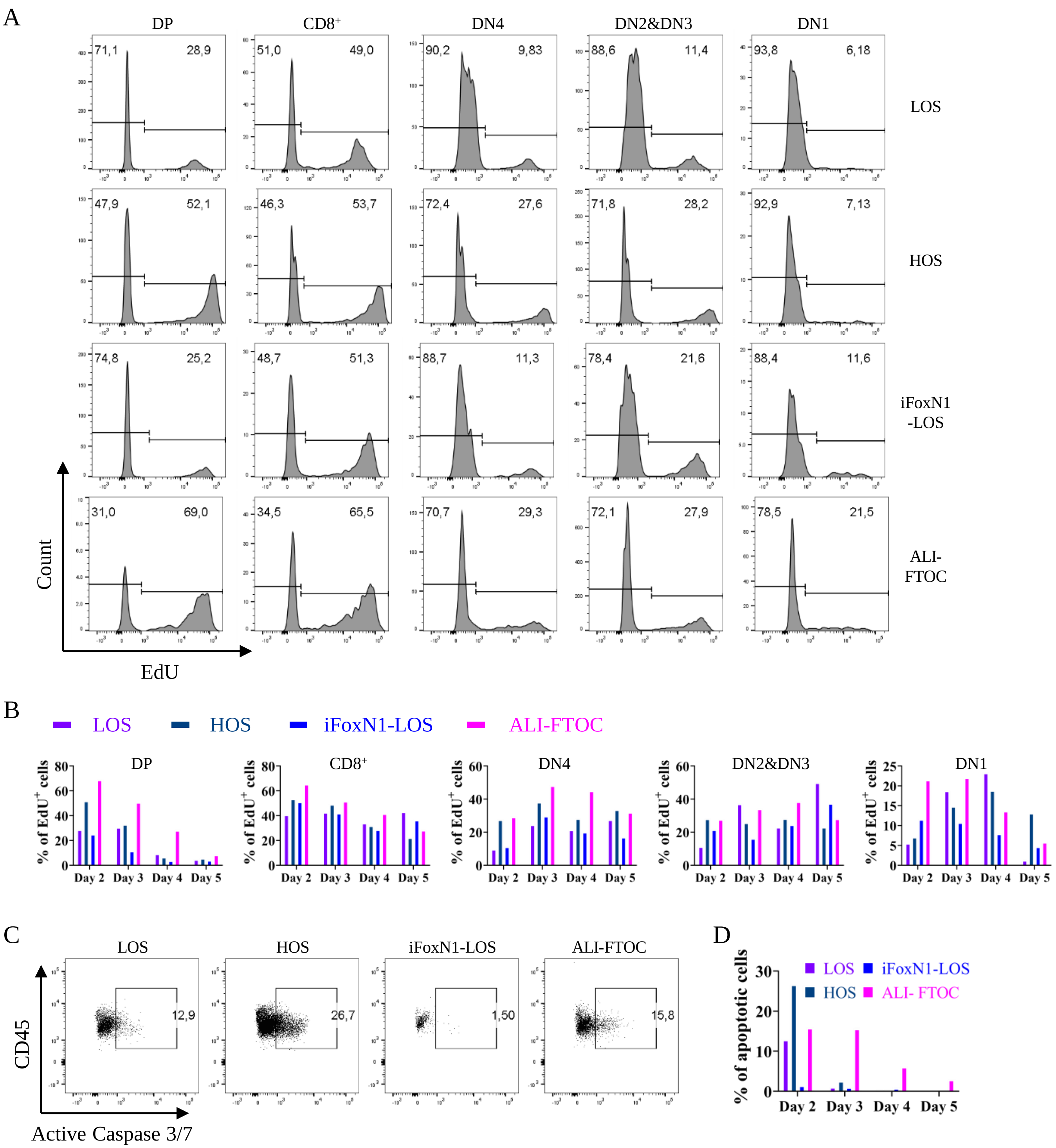

Supplemental Figure 6. Thymocyte proliferation and apoptosis rates. (A) Flow cytometric analysis of thymocyte proliferation rate by EdU incorporation assay. Cells inside of lobes from 4 technical replicate LOS or iFoxN1-LOS cultures, 3 technical replicate HOS cultures, or one ALI-FTOC were pooled together for analysis. Thymus lobes from E14.5 CD1 embryos were used for LOS, HOS, and ALI-FTOC cultures. (B) Column graphs of proliferation rate (%), as indicated by EdU incorporation, of different thymocyte subsets throughout 4 days of cultures. (C) Flow cytometric analysis of thymocyte apoptosis rate by active caspase 3/7 assay of the same samples used above. Cells were pre-gated on $CD45^+$ cells. (D) Column graph of thymocyte apoptosis rate (%), as indicated by cells with active caspase 3/7, throughout 4 days of cultures.

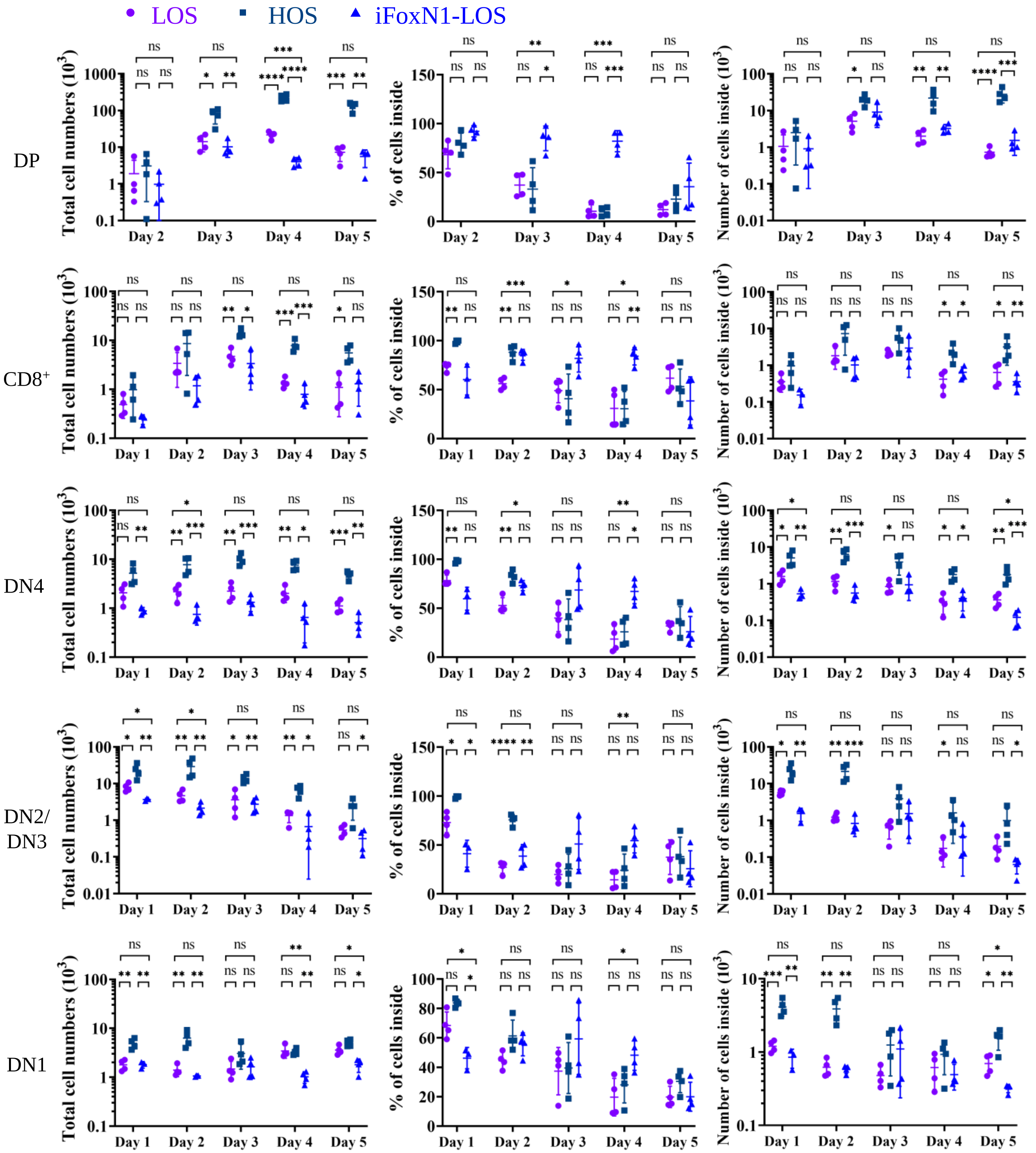

Supplemental Figure 7. Temporal development analysis of different thymocyte subsets from LOS, HOS, and iFoxN1-LOS cultures. Shown are scatter dot plots and statistical analysis (two-way ANOVA with post-hoc Tukey's test) of total cell numbers (left), percentage (middle), and numbers (right) of cells remaining inside of thymus lobes, as indicated. Not significant (ns) $p > 0.05$; *$p < 0.05$; **$p < 0.01$; ***$p < 0.001$; ****$p < 0.0001$.
